# PPAR**γ** in Inflammatory Bowel Disease

**DOI:** 10.1155/2012/620839

**Published:** 2012-09-10

**Authors:** Vito Annese, Francesca Rogai, Alessia Settesoldi, Siro Bagnoli

**Affiliations:** Gastroenterology Unit, Careggi, University Hospital, 50134 Florence, Italy

## Abstract

Peroxisome proliferator-activated receptor gamma (PPAR**γ**) is member of a family of nuclear receptors that interacts with nuclear proteins acting as coactivators and corepressors. The colon is a major tissue which expresses PPAR**γ** in epithelial cells and, to a lesser degree, in macrophages and lymphocytes and plays a role in the regulation of intestinal inflammation. Indeed, both natural and synthetic PPAR**γ** ligands have beneficial effects in different models of experimental colitis, with possible implication in the therapy of inflammatory bowel disease (IBD). This paper will specifically focus on potential role of PPAR**γ** in the predisposition and physiopathology of IBD and will analyze its possible role in medical therapy.

## 1. Introduction

 The peroxisome proliferator-activated receptor gamma (PPAR*γ*) is a nuclear receptor highly expressed in adipose tissue but also intestine, playing a key role in regulation of insulin resistance and inflammation. Recently its role in intestinal diseases, especially colon cancer and intestinal inflammation, is emerging. The discovery that it is the major functional receptor mediating the aminosalicylate activities in inflammatory bowel diseases (IBD) has further enhanced the interest for the role of this receptor in the regulation of gut homeostasis, with possible implication for newer therapeutic targeting. After an extensive search of medical literature in English language from the PubMed database, we aim in this paper to focus on potential role of PPAR*γ* in the predisposition and physiopathology of IBD and to analyze its role in experimental colitis and potential therapy for IBD.

## 2. IBD and PPAR**γ**: Friend or Foe

The inflammatory bowel diseases (IBD), Crohn's disease (CD), and ulcerative colitis (UC) are common causes of gastrointestinal illness characterised by chronic, relapsing intestinal inflammation, often presenting in early childhood [1]. The incidence varies according to geographical location and in Northern Europe IBD may affect upto one in two hundred of the population [2]. The division into CD and UC is made on the basis of clinical, radiological, endoscopic, and histological features. Common clinical features of CD include abdominal pain, diarrhea, weight loss, and fever. Rectal blood loss is not always a feature and up to 10% of patients with CD may not have diarrhea. Inflammatory changes are patchy in distribution and may occur anywhere within the gastrointestinal tract from the mouth to the anus. Approximately 40% of patients with CD will have disease involving both small and large bowel, in 30% the disease is limited to the small bowel, and 27% percent will have colonic disease only. A small minority of patients will have involvement of the more proximal gastrointestinal tract. Inflammation is transmural and histological examination of bowel and lymph nodes will demonstrate epithelioid cell granulomas in 60–70% of cases. In contrast, patients with UC usually present with bloody diarrhea [3]. There may be associated abdominal pain, urgency, and tenesmus. The disease is limited to the mucosal layer of the colon; it will always involve the rectum and may extend proximally in a continuous fashion. 

Current knowledge of aetiology is incomplete, but increasing evidence points towards a combination of environmental triggers in a genetically susceptible individual. More specifically, the intestinal inflammation is thought to result from an inappropriate immune response to microbial antigens of commensal microorganisms [4]. Both diseases manifest themselves primarily in the gastrointestinal tract yet can, in principle, affect all of the organ systems of the body. IBD is also associated with an increased risk of colorectal cancer, which itself is already the third most common cancer in developed countries [2].

The progress in gene discovery in complex disease genetics has increased rapidly in recent years, boosted by the advent of genomewide association (GWA) studies. Few complex diseases have seen as much rapid progress as CD and UC thanks specially to the international inflammatory bowel disease genetics consortium (IIBDGC) who collected around the world some 20,000 cases for each of CD and UC (http://www.ibdgenetics.org/). The statistical power of such large sample sets has proven highly effective in identifying multiple susceptibility loci, even where these confer only modestly increased risk of disease. To date there are 99 IBD susceptibility loci: 71 associated with Crohn's disease, 47 with ulcerative colitis, and 28 with both CD and UC [5, 6]. Amongst these are multiple genes involved in IL23/Th17 signaling (IL23R, IL12B, JAK2, TYK2, and STAT3), genes involved in autophagy, intracellular bacteria processing and innate immunity (NOD2, IRGM, and ATG16L1), and genes involved in barrier (HNF4A, LAMB1, CDH1, and GNA1e). However, from these studies, included the recently reported data with the immunochip from the IIBDGC at DDW 2012 [7], no striking signal of PPAR*γ* gene polymorphisms is emerged, with *P* values of tagging SNPs ranging from 0.005 to 0.01 (personal communication). Poliska et al. have investigated the association of four polymorphisms of PPAR*γ* and IBD; they found haplotypes with both protective and increased risk [8]. Other studies, however, lead to conflicting results [9–13]. Accordingly, a meta-analysis of seven studies with over one thousand UC and CD found no significant association of the Pro12 Ala polymorphism of PPAR*γ* with IBD [14]. 

**Figure 1:**
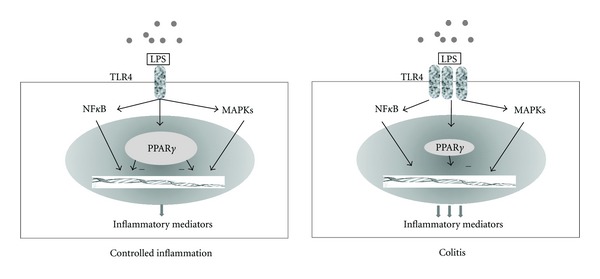
An hypothetical model of influence of PPAR*γ* expression in ulcerative colitis. Induction of PPAR*γ* expression in epithelial cells by bacterial lipopolysaccharide- (LPS-) activated TLR4, in turn leads to break NF*κ*B and MAPK pathways to produce inflammatory mediators. The reduced expression of PPAR*γ* together with TLR4 upregulation might enhance the inflammatory mediators production thus resulting in mucosal damage.

In contrast, PPAR*γ* is highly expressed in colonic epithelial cells and to a lesser degree into macrophages and lymphocytes [15]. In addition, its expression in the colon is closely linked to intestinal-microbial interaction. Using quantitative PCR, western blot, and immunohistochemical assay, a 60% decreased expression of PPAR*γ* was observed at the mRNA and protein levels in the colon of UC patients, compared with CD and controls [16]. This impaired expression was found in both inflamed and noninflamed areas and limited to epithelial cells, suggesting that this modified expression is not secondary to the inflammatory process (Figure 1). A possible explanation is the occurrence of epigenetic changes [16]; this hypothesis is corroborated by the demonstration of similar levels of PPAR*γ* in peripheral mononuclear cells of IBD patients and controls and lack of significant polymorphisms of PPAR*γ* in UC patients. Another intriguing possibility is that the Toll-like receptor 4 (TLR4) signaling to PPAR*γ* is impaired in UC. The resulting imbalance between elevated levels of TLR4 and reduced expression of PPAR*γ* may lead to loss of mucosal tolerance to luminal LPS, resulting in mucosal inflammation [16]. In contrast, Yamamoto-Furusho et al. reported that the mRNA PPAR*γ* expression was significantly reduced in the mucosa with active UC compared to the mucosa of patients in remission, with a significant correlation with disease activity [17]. 

 More recently, another important role of PPAR*γ* in the modulation of intestinal inflammation has been put forward. In healthy individual, immune cells and gut mucosa remain largely inactive towards 10^14^ bacteria of the intestinal microflora. This tolerance is attributed to the prominent presence of regulatory immune cells that may be triggered by the resident microflora and whose function is antagonistic to inflammatory pathways stimulated by pathogenic bacteria [18]. The effector cells are M1 macrophages and dendritic (De) cells secreting inflammatory mediators including factors stimulating additional resting macrophages, dendritic cells precursors (monocytes), and T cells. De and M1 present antigen to resting T cells while secreting cytokines (IL12, IFN-*γ*, TNF-*α*, and IL-23) and induce the differentiation to proinflammatory T-helper, specifically Th1 and Th17. The immune response kills the invading bacteria, but may also cause indiscriminate tissue damage. In sterile organ systems, the inflammatory process usually ceases once the antigen population is eliminated. However, in the gut because of the resident microflora, the antigen population cannot be eliminated and the mounted inflammation could be more harmful for the host than the invading bacteria itself, for example, increasing gut permeability and infiltration of bacteria in the lamina propria. In healthy individuals, the gut mucosa contains various regulatory factors such as M2 macrophages, tolerogenic dendritic cells (Dt), and T regulatory cells. This regulatory pathway, by binding to ligands recognized as self to specific receptors, induces the differentiation and switches from M1 to M2 and from De to Dt. One such receptor is PPAR*γ* expressed in T cells, dendritic cells, macrophages, and epithelial cells [19, 20].

## 3. PPAR**γ**: Structure, Function, and Expression in the Gut

 PPAR*γ* belongs to the nuclear receptor family consisting of approximately 50 transcription factors implicated in many biological function. It is an essential nuclear receptor controlling the expression of a large number of regulatory genes in lipid metabolisms, insulin sensitization, inflammation, and cell proliferation [48].

Similarly to other nuclear hormone receptors, PPAR*γ* displays a central DNA-binding domain, a C-terminal ligand-binding domain, and two transcription-activation function motifs (AF-1 and AF-2) [49]. Binding of ligands to PPAR*γ* leads to a conformational change in the receptor which allows recruitment of co-activator proteins to then induce transcriptional activation. The transcriptional activity of PPAR*γ* is regulated by post-translational changes such as phosphorylation or ubiquitination. The activation requires heterodimerization within the nucleus with another nuclear receptor named retinoid X receptor *α* (RXR*α*), leading to bind a specific DNA sequence elements known as peroxisome proliferator elements (PPREs) [50]. PPAR*γ* interferes with inflammatory pathways by interactions with transcription factors such as nuclear factor kappa B (NF-*κ*B), activating protein-1 (AP-1), signal transducer and activator of transcription (STAT), and nuclear factor-activated T cell (NFAT). For example, PPAR*γ* is able to form a complex with the NF-*κ*B subunit p65 at a nuclear level and this complex is exported from the nucleus leading to an altered expression of proinflammatory NF-*κ*B-mediated gene expression. Inhibition of NF-*κ*B in response to the activity of PPAR*γ* ligands attenuates the expression of various cytokines in colonic epithelial cells such as IL-1*β*, COX-2, IL-6, IL-8, TNF-*α*, INF-*γ*, iNOS, and chemokines [51, 52]. Its expression has been initially investigated in adipose tissue where it plays a key role in adipocyte differentiation and insulin responses. More recently the colon has been found to highly express PPAR*γ* in epithelial cells but also macrophages and lymphocytes [16, 52, 53]. Regulation of expression is incompletely understood; in vivo mRNA expression is negatively influenced by a long-term hypocaloric diet and fasting and positively by obesity and a rich in fatty acids diet. More recently a close link between intestinal microbial flora and PPAR*γ* expression has been demonstrated. The stimulation of expression is probably multifactorial and involves the LPS recognition by the toll-like receptor (TLR), especially LPS of gram-negative bacteria and TLR4. Another alternative way of stimulation is the production through the bacteria of volatile fatty acid butyrate [15].

## 4. Experimental Model of Colitis

The initial evidence of the involvement of PPAR*γ* in the regulation of intestinal inflammation derives from the observation of the use of PPAR*γ* synthetic agonist thiazolidinedione (TZD) in mice dextran sodium sulfate- (DSS-) induced colitis. In the study by Su CG et al., both troglitazone and rosiglitazone dramatically reduced the colonic inflammation in mice and in addition significantly attenuated cytokine gene expression in colon cancer cell lines through NF-*κ*B inhibition [21]. This first evidence was subsequently confirmed in another model of experimental colitis induced in mice by intrarectal administration of 2,4,6-trinitrobenzene sulfonic acid (TNBS) [51]. TZD given preventively significantly reduced mortality, severity of macroscopic and histological lesions, and markers of inflammation. So far several studies have reported similar prophylactic and therapeutic efficacy of PPAR*γ* agonists in different animal models (mice, rats, and pigs) with different models of colitis induced by chemical compounds [22–34], ischaemia [35–38], bacteria [39], or genetically modified animals [43–47, 65, 66] (Table 1) [40–42]. Moreover a beneficial effect of PPAR*γ* ligands has been demonstrated in colon carcinogenesis. Of interest, the use of probiotics (VSL#3), conjugated linoleic acid, n-3 polyunsaturated fatty acids, cannabidiol, punic acid, *α*-eleostearic acid, and a polyphenolic compound has prove beneficial effect on animal model of intestinal inflammation through the activation of PPAR*γ* [67] (Table 1).

Taken together lessons from animal studies suggest that: (a) natural and synthetic PPAR*γ* ligands are both effective in the treatment of acute and chronic animal models of inflammation; (b) the prophylactic effect is more pronounced than the therapeutic effect; (c) the therapeutic effect is apparently dependent by the abundance of PPAR*γ* in the target tissue as demonstrated by the genetically modified animals. This information translated into clinical ground could suggest a major role of PPAR*γ* agonists in maintenance rather than induction of remission in IBD patients. Moreover, with PPAR*γ* being expressed not only in the epithelial cells but also in macrophages, T, and B cells, more investigations are needed to disclose which cell type expression of PPAR*γ* is more crucial for the potential therapeutic effect. 

## 5. Dietary Modulation of PPAR**γ**

 A large number of dietary nutrients are able to modulate PPAR*γ* (see Table 2). Fatty acids and their metabolites can affect gene expression by binding to PPAR*γ*. The effect of n-3 PUFAs is well documented; linoleic acid is the major PUFA in human diet and several derivatives like conjugated linoleic acid (CLA), nitrolinoleic acid, and gamma linoleic acid have shown activation property on PPAR*γ* [54, 55]. Another fatty acid-derived metabolite known to be a strong PPAR*γ* inducer is the prostaglandin 15d-PGJ2 as demonstrated in several animal models [36]. Glutamine is the preferential substrate of enterocytes and is considered essential in stress situations. In a rodent model of ischemia reperfusion, glutamine also acted as PPAR*γ* agonist, as protective effect was abrogated by a PPAR*γ* inhibitor [38]. Various spicy foods such as curcumin and capsaicin have been shown to activate PPAR*γ*. The anti-inflammatory property of curcumin is also expressed by the inhibition of NF-*κ*B, but is clearly blocked by PPAR*γ* inhibitor [56–58]. Also ginsenosides, compounds derived by ginseng, may have opposite effects being ginsenoside 20S a strong inducer and Rh2 an inhibitor of PPAR*γ* [59, 60]. Finally, other inducers are flavonoids, epigallocatechingallate derived from green tea, resveratrol derived from grapes and wine, butyrate, and micronutrients such as vitamin E and selenium [61–64, 72] (Table 2). 

## 6. PPAR**γ** and Therapy of Ulcerative Colitis

5-ASA is one of the oldest anti-inflammatory agents used for treatment of IBD, although the mechanism underlying its effects is still unknown. It is the mainstay of therapy for the majority of patients with UC for the induction of remission, maintenance, and possibly chemoprevention of colorectal cancer [73]. Recently, functional, biological, pharmacological, and chemical evidence has shown that aminosalicylates are a new functional synthetic ligand for PPAR*γ* in colonic epithelial cells [33]. PPAR*γ* is indeed the key receptor mediating the 5-ASA activity, by trans-repressing several key target genes such as nuclear factor *κ*B, signal transducers, and activators of transcription. 

 Since in animal models treatment with PPAR*γ* ligands has been demonstrated to attenuate inflammatory cytokines production such as IL-1*β* and TNF-*α*, it has been hypothesized the use of PPAR*γ* ligands, like thiazolidinedione (TZD), in the therapy of UC [15]. One potential candidate is rosiglitazone, an antidiabetic drug. A first open-label pilot study in 15 patients with mild to moderate UC refractory to 5-ASA has evaluated the efficacy of the PPAR*γ* ligand rosiglitazone (4 mg orally twice daily) (Table 3). These patients were refractory to conventional treatment, including corticosteroids and immune modulators. After 12 weeks of treatment, a striking reduction in disease activity index score was reported, with clinical and endoscopic remission in 27% and 20% of patients, respectively [68]. Liang and Quayang performed a clinical trial in China in 42 patients with mild to moderate UC [69]. Patients were allocated alternatively to the treatment of rosiglitazone 4 mg/day plus 5-ASA 2 gr or sulfasalazine 3 gr, while the control group received 5-ASA or sulfasalazine alone for 4 weeks. The remission rate was greater in the rosiglitazone group (71.4% versus 57.1%), with a significant improvement of the histologic score (*P* < 0.05). Moreover in the treatment group the PPAR*γ* expression was increased compared to baseline [69]. 

Recently, a randomized multicenter double-blind, placebo-controlled trial has been published by using rosiglitazone 4 mg orally twice daily versus placebo for 12 weeks in 105 patients with mild to moderate ulcerative colitis [70]. Disease activity was measured by Mayo score with a primary endpoint of a clinical response (≥2 points reduction) at week 12, while clinical remission, endoscopic remission, and quality of life changes were considered secondary outcomes. After 12 weeks of therapy, 23 patients (44%) treated with rosiglitazone and 12 patients (23%) treated with placebo achieved clinical response (*P* = 0.04). Remission was achieved in 9 patients (17%) treated with rosiglitazone and 1 patient (2%) of the placebo arm (*P* = 0.01). However, endoscopic remission was uncommon in either arms (8% versus 2%; *P* = 0.34). Clinical improvement was clearly evident already at 4 week (*P* = 0.049), while quality of life was significantly improved at week 8 (*P* = 0.01), but not at week 4 and 12. The safety profile was remarkably safe, with adverse events occurring at similar rates in both groups; in particular edema and weight gain, as expected, were more common in the rosiglitazone group. Of interest, no cases of symptomatic hypoglycemia were reported. 

Pederson and Brynskov reported the use of rosiglitazone enema compared to mesalazine in fourteen patients with distal UC [71]. Rosiglitazone had a similar effect compared to mesalazine enema, with a significant reduction of Mayo score (*P* < 0.01). In addition rosiglitazone restored the PPAR*γ* activity in the inflamed area which was fourfold reduced before treatment compared with noninflamed areas and controls. 

Although substantial research has focused on potential anti-inflammatory effects of TZD PPAR*γ* ligands, their mechanism of action, particularly in the colon, is not well defined. The 5-ASA compounds largely used in UC are able to bind to PPAR*γ* [33]. In the study of Lewis et al. [70] the majority of patients were on concomitant therapy with 5-ASA. Since rosiglitazone has a higher affinity to PPAR*γ* compared to 5-ASA, one possible explanation of the efficacy is a more powerful stimulation and anti-inflammatory property of PPAR*γ*. Alternatively, the effect could be mediated at the mucosal level, where the PPAR*γ* is largely expressed [74]. Of note, large clinical trials with rosiglitazone in the treatment of psoriasis, another inflammatory disease, did not demonstrate efficacy, thus suggesting a “topical” and not a systemic effect in patients with UC [75].

Being also involved in cell proliferation, apoptosis, and modulation of cytokine production with antitumorigenic effect, PPAR*γ* is also extremely important for the basis of chemoprevention strategies against colorectal cancer. For these reasons, there is an active ongoing research to disclose and investigate safer PPAR*γ* agonists, with topical effect and direct targeting of the colon, possibly void of metabolic and systemic effect. 

## 7. PPAR**γ** and Therapy of Crohn's Disease

Recent data have suggested that the role of PPAR*γ* in IBD physiopathology is not limited to UC but might involve also CD. Based on SAMP1/YitFc animal example, developing a spontaneous ileitis due to a defect of expression of PPAR*γ* in ileal crypts, the polymorphisms of PPAR*γ* has been tested in CD. Sugawara et al. [44] demonstrated that two intronic SNPs exhibited a significant lower frequency in CD compared to controls. However, these findings have not been independently replicated yet. Moreover, no data are available by using PPAR*γ* ligands in medical therapy of CD, in which 5-ASA compounds are generally believed to be of little or no efficacy [73]. 

## 8. Conclusion and Take-Home Messages

PPAR*γ* receptors are widely and highly expressed in the colon, being a key regulator factor of bacteria-induced mucosal inflammation. Moreover, they are directly involved in the mechanism of action of mesalazine, which is largely used and effective in UC. In addition, they are involved in the process of tumor suppression, especially in the colon. Therefore, beside the potential interest in the IBD physiopathology and genetic predisposition which is still under evaluation, it is highly expected that new molecules specifically targeting the intestinal receptors and void of action in the adipose tissue and insulin action could be developed and tested. Several tens of compounds have been already synthetized, some with 30–50-fold higher affinity against PPAR*γ* and potentially higher efficacy than 5-ASA. These compounds are not that far from clinical application with potential implication in controlling the inflammation, better handling of host-bacterial interactions, and possible chemoprevention. In addition, a better understanding of the role of microbiota on PPAR*γ* receptors should be elucidated, since some commensal bacterial or natural ligands of foods may directly activate and increase the expression of PPAR*γ*, thus determining a “biologic” anti-inflammatory action.

## Figures and Tables

**Table 1:** Anti-inflammatory properties of PPAR*γ* agonists in experimental models.

Model	PPAR*γ* modulator	Effect	Authors
Acute colitis			

DSS	Troglitazone	↓ Colonic inflammation	Su et al. 1999 [21]
		↓ Cytokine gene expression	
	Rosiglitazone	Reduced inflammation	Saubermann et al. 2002 [22]
		More severe colitis	Ramakers et al. 2007 [23]
	Pioglitazone	Prevention colitis	Takagi et al. 2002 [24]
		Recovery from colitis	Hontecillas et al. 2011 [25]
		Reduced CXCL10 level	Schaefer et al. 2005 [26]
	PUFA	Accelerated remission	
	CLA	Delayed onset of colitis	Bassaganya-Riera 2006 [27]
	CLA + VSL#3	Improvement of colitis	Bassaganya-Riera et al. 2012 [28]
	*α*-Eleostearic acid	Improvement of colitis	Lewis et al. 2011 [29]

TNBS	Troglitazone	Reduced inflammation	Desreumaux et al. 2001 [30]
	Rosiglitazone	Reduced inflammation	
		Reduced inflammation	Sànchez-Hidalgo et al. 2007 [31]
	Pioglitazone	Reduced CXCL10 level	Schaefer et al. 2005 [26]
	FMOC-L-leu	Reduced inflammation	Rocchi et al. 2001 [32]
	5-ASA	Reduced inflammation	Rousseaux et al. 2005 [33]
	5-ASA in PPAR*γ*+/−	No efficacy of 5-ASA	

Acetic acid ischaemia	THSG	Attenuated colon lesions	Zeng et al. 2011 [34]
	Rosiglitazone	Protection	Nakaijma et al. 2001 [35]
	15-d-PGJ2	Reduced injury	Cuzzocrea et al. 2003 [36]
	NS-398	Protection	Sato et al. 2005 [37]
	Glutamine	Protection	Sato et al. 2006 [38]

Bacterial	CLA	Attenuated inflammation	Hontecillas et al. 2002 [39]

Chronic colitis			

DSS	Triglitazone	↓Cell proliferation	Tanaka et al. 2001 [40]
TNBS	Rosiglitazone	Protection	Sànchez-Hidalgoet al. 2005 [41]
CD4-CD45RBhi	CLA	Reduced inflammation	Bassaganya-Riera et al. 2004 [42]
IL-10 KO	Rosiglitazone	Slow onset colitis	Lytle et al. 2005 [43]
SAMP1/Yirfc	Rosiglitazone	Decreased severity	Sugawara et al. 2005 [44]

Genetic models			

PPAR*γ* +/−			Desreumaux et al. 2001 [30]
	Ischaemia	More severe damage	Nakaijma et al. 2011 [35]
			Saubermann et al. 2002 [22]
		
	DSS + PUA	Loss protective effect PUA	Hontecillas et al. 2011 [25]

AdPPAR*γ*			Katayama et al. 2003 [39]

SAMP1/yifc			Sugawara et al. 2005 [44]

PPAR*γ*Cre+			Bassaganya-Riera et al. 2004 [42]

PPAR*γ*ΔM*φ*	DSS	Increased susceptibility	Shah et al. 2007 [45]

PPAR*γ*fifi	DSS	Accelerated colitis	Guri et al. 2010 [46]

		Worsen colonic lesions	Mohapatra et al. 2010 [47]

5-ASA: 5-aminosalycilic acid; 15dPGJ2: 15-deoxy-Δ12,14-prostaglandin J2; CLA: conjugated linoleic acid; PUFA: n-3 polyunsaturated fatty acids; DSS: dextransodiumsulphate; FMOC-L-leu: fluorenylmethyloxycarbonyl-L-leucine; IL-10 KO: interleukin 10 knockout mice; PPAR*γ*Cre: PPAR*γ* conditional knockout mice; TNBS: 2,4,6-trinitrobenzene sulfonic acid; PUA: punicic acid; THSG: 2,3,5,4′-tetrahydroxystilbene-2-O-beta-D-glucoside.

**Table 2:** Nutrients with demonstrated anti-inflammatory effects mediated through PPAR*γ*.

Nutrient	Dietary source	Models	Authors
*α*-linoleic acid	Green vegetables, flax	Intestinal epithelial cells	Marion-Letellier 2008 [54]
Docosahexaenoic	Fish	Intestinal epithelial cells	Marion-Letellier 2008 [54]
Eicosapentaenoic ac.			
Conjugated linoleic acid	Beef, bovine milk	Intestinal epithelial cells DSS colitis	Allred et al. 2008 [55]
Glutamine	Beef, chicken, fish	Ischaemia reperfusion	Sato et al. 2006 [38]
Curcumin	Tumeric powder	TNBS colitis	Salh et al. 2003, Deguchi et al. 2007 [56]/[57]
Capsaicin	Cayenne pepper	Intestinal epithelial cells	Kim et al. 2004 [58]
Ginsenoids	Ginseng	Adypocites	Han et al. 2006, Hwang et al. 2007 [59]/[60]
Resveratrol	Grapes, wine, peanuts	Intestinal epithelial cells	Morikawa et al. 2007 [61]
Butyrate	Unabsorbed carbohydrate	Intestinal epithelial cells	Schwab et al. 2007 [62]
Vitamin E	Nuts, seeds, oils	Colon cancer cell lines	Campbell et al. 2003 [63]
Selenium	Plant foods	Macrophages	Vunta et al. 2007 [64]

**Table 3:** Efficacy of rosiglitazone therapy in ulcerative colitis (*P values < 0.05).

Authors	N° pts	Study design	Treatment	% Efficacy		
				Response	Remission	Mucosal healing
Lewis et al. 2001 [68]	15	Open 12 weeks	4 mg tid	—	27	20
Liang and Ouyang 2006 [69]	42	Random versus 5-ASA	4 mg	—	71 versus 57*	—
Lewis et al. 2008 [70]	105	12 wks versus Plac	4 mg tid	44 versus 23*	17 versus 2*	8 versus 2
Pederson and Brynskov 2010 [71]	14	Open versus 5-ASA	4 mg versus 1 enema	= 5-ASA	= 5-ASA	—
